# Lamin A/C and the Immune System: One Intermediate Filament, Many Faces

**DOI:** 10.3390/ijms21176109

**Published:** 2020-08-25

**Authors:** Angela Saez, Beatriz Herrero-Fernandez, Raquel Gomez-Bris, Beatriz Somovilla-Crespo, Cristina Rius, Jose M. Gonzalez-Granado

**Affiliations:** 1 Centro de Biotecnología y Genómica de Plantas (CBGP), Instituto Nacional de Investigación y Tecnología Agraria y Alimentaria (INIA), Campus de Montegancedo, Pozuelo de Alarcón, Pozuelo de Alarcón, 28223 Madrid, Spain; angela.saez@upm.es; 2 LamImSys Lab, Instituto de Investigación Hospital 12 de Octubre (imas12), 28041 Madrid, Spain; beatriz.herrero@uam.es (B.H.-F.); rgomez.imas12@correo.h12o.es (R.G.-B.); beatriz.somovilla.imas12@h12o.es (B.S.-C.); 3 Departamento de Fisiología, Facultad de Medicina, Universidad Autónoma de Madrid (UAM), 28029 Madrid, Spain; 4 Faculty of Biomedical and Health Sciences, Universidad Europea de Madrid (UEM), Villaviciosa de Odón, 28670 Madrid, Spain; cristina.rius@universidadeuropea.es; 5 Centro Nacional de Investigaciones Cardiovasculares (CNIC), 28029 Madrid, Spain; 6 CIBER de Enfermedades Cardiovasculares, 28029 Madrid, Spain

**Keywords:** lamin A/C, macrophage, neutrophil, dendritic cell (DC), T cell, cancer, viral infection, *Leishmania*, inflammatory bowel disease

## Abstract

Nuclear envelope lamin A/C proteins are a major component of the mammalian nuclear lamina, a dense fibrous protein meshwork located in the nuclear interior. Lamin A/C proteins regulate nuclear mechanics and structure and control cellular signaling, gene transcription, epigenetic regulation, cell cycle progression, cell differentiation, and cell migration. The immune system is composed of the innate and adaptive branches. Innate immunity is mediated by myeloid cells such as neutrophils, macrophages, and dendritic cells. These cells produce a rapid and nonspecific response through phagocytosis, cytokine production, and complement activation, as well as activating adaptive immunity. Specific adaptive immunity is activated by antigen presentation by antigen presenting cells (APCs) and the cytokine microenvironment, and is mainly mediated by the cellular functions of T cells and the production of antibodies by B cells. Unlike most cell types, immune cells regulate their lamin A/C protein expression relatively rapidly to exert their functions, with expression increasing in macrophages, reducing in neutrophils, and increasing transiently in T cells. In this review, we discuss and summarize studies that have addressed the role played by lamin A/C in the functions of innate and adaptive immune cells in the context of human inflammatory and autoimmune diseases, pathogen infections, and cancer.

## 1. Lamin A/C

The mammalian nuclear envelope separates the nucleoplasm from the cytoplasm and is composed of two lipid bilayers: the outer and inner nuclear membranes, nuclear pore complexes, and the nuclear lamina [1]. The outer nuclear membrane (ONM) is continuous with the endoplasmic reticulum, whereas the inner nuclear membrane (INM) surrounds the nuclear lamina [1]. The nuclear lamina is a dense fibrous protein meshwork mainly composed of type V intermediate filament proteins called lamins; these are closely connected to a variety of INM-associated proteins and interact with portions of the chromatin [1]. Lamins can be categorized as A-type or B-type, based on their primary sequence and biological properties [2]. In mammals, lamins are encoded by three genes, i.e., *LMNB1* encodes lamin B1, *LMNB2* encodes lamin B2 and lamin B3, and *LMNA* encodes the major forms lamin A and C (referred to as lamin A/C in this manuscript), as well as lamins AΔ10 and C2 [1,3,4].

Lamin A/C contributes to nuclear mechanical stability, nuclear structure maintenance, and nuclear positioning, and mediates higher-order chromatin organization, epigenetic regulation, nuclear pore complex organization, gene transcription, nuclear envelope breakdown, and reassembly during mitosis, DNA replication, DNA damage response, cell cycle progression, cell differentiation, and cell polarization during migration [1,5,6,7]. These functions have been investigated in diverse cell types, but only a few studies have been performed on immune cells. In this review, we summarize the role of lamin A/C in immune system-mediated cellular mechanisms and its importance in some immune system-associated human diseases.

## 2. Immune System

The immune system is composed of two major arms: innate and adaptive immunity. Innate immunity is mediated by myeloid cells, which generate a rapid and nonspecific response as a first line of defense. Innate immune cells express pattern recognition receptors (PRRs) such as toll-like receptors (TLRs), allowing them to recognize pathogen-associated molecular patterns (PAMPs) and damage-associated molecular patterns (DAMPs). Innate immune cells mediate host defense and inflammation by producing cytokines and chemokines, activating the complement cascade and phagocytosis, or activating adaptive immunity by presenting antigens. Notable cells of the innate immunity include neutrophils, macrophages, and dendritic cells (DCs) [8,9,10].

Specific adaptive immunity is activated by antigen presentation by antigen presenting cells (APCs) and the cytokine microenvironment, and is mainly mediated by the cellular function of CD4 and CD8 T cells and the production of antibodies by B cells. Other cytotoxic cells, such as natural killer T cells (NKT cells) and γδ T cells, are at the border between innate and adaptive immunity [8,9,10].

## 3. Lamin A/C Expression in Immune Cells

**Figure 1:**
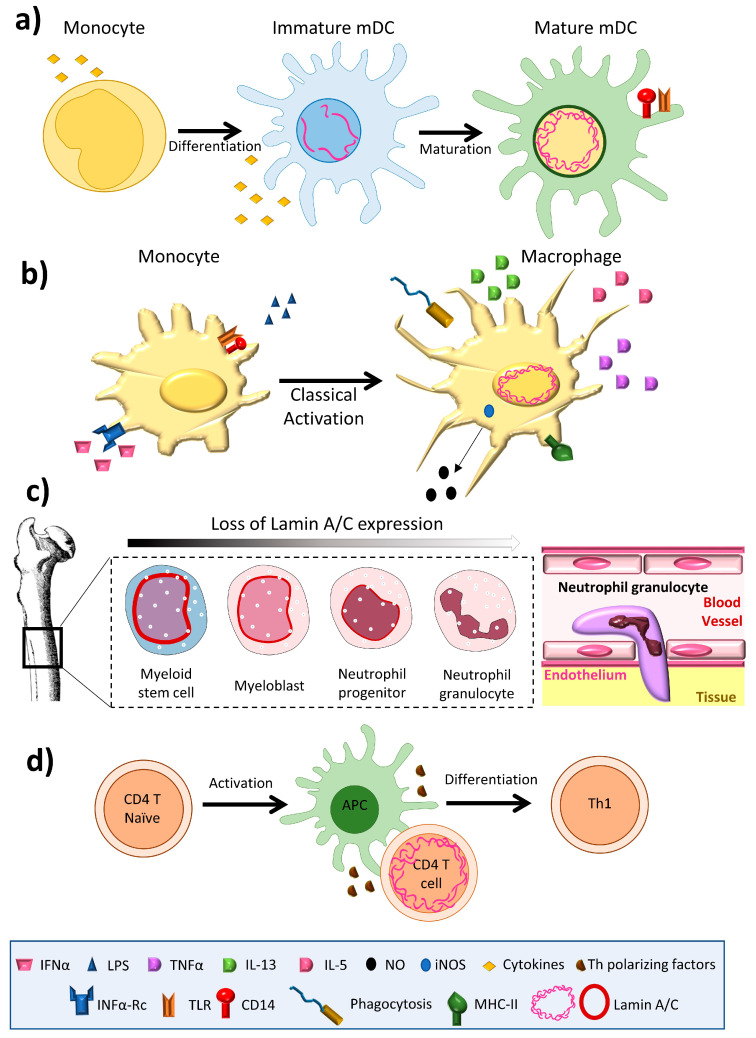
Lamin A/C levels are finely regulated in immune cells. (**a**) Dendritic cells have an intermediate lamin A/C content, between that of neutrophils and macrophages, which is associated with intermediate viability and migration. (**b**) Macrophages increase lamin A/C content upon differentiation and activation. (**c**) During granulopoiesis, neutrophil precursors change their round nuclear shape for a characteristic lobed nucleus, a process linked to almost complete loss of lamin A/C expression and augmented expression of the lamin B receptor (LBR). Neutrophil loss of lamin A/C enables them to pass through narrow spaces. (**d**) T cells show a transient peak in lamin A/C expression upon recognition of an antigen presented by an antigen presenting cell.

Lamin A/C is abundantly expressed in most differentiated cells, but is absent or infrequently expressed in pluripotent stem cells and embryos during early development [11]. The amount of lamin A/C in interphase of somatic cells is quite stable, exhibiting slow subunit exchange [4]; its expression has thus been linked to cell differentiation [12]. Aging is associated with small changes in the amount of lamin A/C in osteoclasts [13]. The amount of lamin A/C varies greatly between immune cell types, with macrophages and dendritic cells expressing high levels [14,15], but inactivated T and B cells expressing barely detectable amounts [16,17] (Figure 1). Remarkably, unlike most other somatic cells, immune cells have been shown to undergo very rapid changes in lamin A/C protein level during differentiation, activation, or migration [16,17,18,19].

Among innate immune cells, high lamin A/C mRNA expression has been reported in human monocyte-derived dendritic cells [20], and high protein expression is observed in rat bone marrow derived dendritic cells [14] (Figure 1a) and macrophages [15,21,22,23] (Figure 1b). Serum-free differentiation of rat and human macrophages was accompanied by increased expression of lamin A/C [14]. Lamin A/C is also expressed in thyoglycolate-induced mouse peritoneal macrophages and the mouse monocyte/macrophage-like cell line J774A.1 [21,24]. Lamin A/C expression increases during the differentiation of human peripheral-blood monocytes into macrophages [22]. Human promyelocytic leukemia HL-60 cells can be induced to differentiate in vitro into monocytes/macrophages with phorbol ester (TPA) or into granulocytes with retinoic acid. HL-60 cells contain 0.1 × 10^6^ copies of lamin A/C per cell [25]. Phorbol ester-induced HL-60 differentiation to the macrophage phenotype induces lamin A/C expression [25,26] and promotes the redistribution of lamin A to the nuclear periphery [23]. In contrast, differentiation to neutrophil granulocytes is associated with lamin A/C downregulation [27]; human primary neutrophils express very low levels of lamin A/C [28], and retinoic acid differentiation of HL-60 cells into neutrophils results in a downregulation of lamin A/C expression [29,30] (Figure 1c). Macrophage-differentiated HL-60 cells thus contain higher amounts of lamin A/C than neutrophil-type cells [30,31] (Figure 1b,c).

In the adaptive immune system, lamin A/C expression is undetectable in resting B lymphocytes from human blood [17] and mice [32], but increases upon treatment with lipopolysaccharide (LPS) [17]. Lamin A/C has been detected in some B cell lymphomas [17,33], but not in unstimulated human and mouse T lymphocytes [34,35]; it is present in only very few lymphocytes from rat bone marrow cultures [21]. However, lamin A/C has been detected in activated human peripheral blood lymphocytes, CD4+ T lymphocytes, and in CD30+ lymphoid cells [33,36,37]. In line with this evidence, although very few resting human and mouse T cells express lamin A/C, T cell activation by antigen recognition or another TCR-dependent stimulus triggers a transient and potent increase in lamin A/C mRNA and protein expression [16]. In line with this finding, activation of peripheral blood mononuclear cells with the plant lectin concanavalin A results in a sharp increase in the percentage of lamin A/C positive cells [38], and lamin A/C expression is also potentiated during phorbolester-mediated differentiation of HL-60 cells into macrophage-like cells [23].

Little is known about the mechanisms regulating lamin A/C expression in immune cells. One candidate is the Akt/PKB signaling pathway, which is induced in immune cells, for example upon T cell activation [39], and regulates pre-lamin A transcription during interphase in other cell types, possibly via the transcription factors FoxO, Sp1/Sp3, AP1, and CREB [40]. The peak in lamin A/C expression upon T cell activation is followed by a sharp decline [16], which may be the result of diminished de novo synthesis or increased elimination of lamin A/C proteins. Another possibility is that lamin A/C expression in immune cells is controlled by microRNA. Brain expression of lamin A, but not lamin C, is regulated by the microRNA miR-9 [41]. Moreover, a retinoic acid (RA) responsive element has been found in the LMNA promoter of P19 embryonic carcinoma cells [42], and RA reduces lamin A/C expression in HL-60 cells [30] and human monocyte-derived myeloid cells [43]. In T cells, RA binding to RARα or RARγ reduces antigen-recognition-induced lamin A/C expression in CD4+ T cells in vitro [44]. Moreover, the lamin A/C content in CD4+ T cells upon antigen recognition depends on the specific T cell microenvironment in vivo; lamin A/C is highly expressed in activated CD4+ T cells located in peripheral lymph nodes, whereas expression is significantly lower in activated CD4+ T cells in mesenteric lymph nodes and spleen [44]. This effect correlates with known tissue-microenvironment-dependent variations in RA disposal [45]. CD103+ DCs in the gut, mesenteric lymph nodes, and Peyer’s patches can release RA to T cells undergoing activation [46], whereas CD103- DCs, located mainly in peripheral lymph nodes, do not produce RA [47].

## 4. Lamin A/C in Innate Immunity

### 4.1. Neutrophils

Neutrophils (also called polymorphonuclear leukocytes) are the first line of defense against infections and an essential cell type in the initiation of the inflammatory response [48]. Deficiencies in the migration, activation, and survival of neutrophils are implicated in the origin of many inflammatory diseases, including acute lung injury following major trauma and sepsis and chronic inflammation in diseases such as chronic obstructive pulmonary disease (COPD) and inflammatory arthritis [49,50,51].

The bone marrow of a healthy adult produces 10^11^ neutrophils daily, with each cell surviving for a few hours in the circulation [52,53,54]. Upon infection or tissue damage, neutrophils rapidly migrate from the circulation into tissues in response to chemoattractants such as CXCL8 (IL-8). At the same time, blood neutrophils are replaced from the bone marrow. The migration of neutrophils across the endothelium is mediated through interactions between integrin α9β1, expressed on neutrophils, and VCAM-1, expressed on the activated endothelium. During this recruitment, the lifetime of neutrophils is increased by their stimulation with GM-CSF [55]. Neutrophils are activated upon recognition of PAMPs or DAMPs, and contribute to the resolution of infection through phagocytosis, the release of granules and ROS, and the formation of neutrophil extracellular traps (NETs) [56]. Moreover, neutrophils produce several cytokines that promote the recruitment and activation of other leukocytes and stimulate tissue recovery [48].

**Figure 2:**
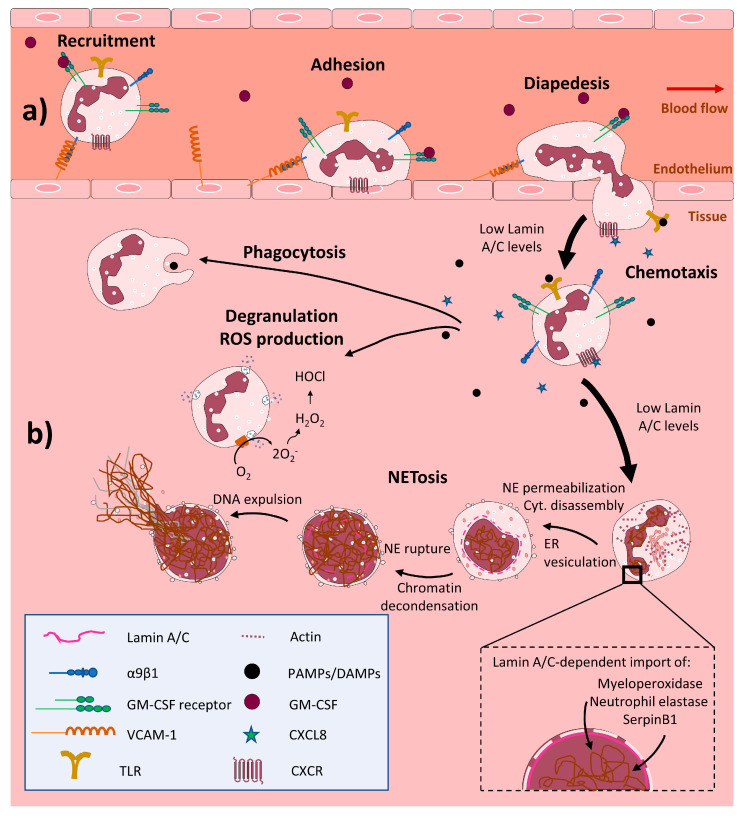
Low lamin A/C expression in neutrophils enables them to pass through narrow spaces during transendothelial migration. Upon infection or tissue damage, neutrophils are rapidly recruited from the circulation to tissues in response to chemoattractants such as CXCL8. The transmigration of neutrophils across the endothelium is mediated by interaction between integrin α9β1 expressed on neutrophils and VCAM-1 expressed on the activated endothelium. Stimulation of neutrophils by GM-CSF during recruitment augments their lifespan. Neutrophils recognize PAMPs or DAMPs through the activation of TLRs and contribute to the resolution of infection through phagocytosis, the release of granules and ROS, and the formation of neutrophil extracellular traps (NETs) NETosis. Neutrophils engage several cellular mechanisms during the generation of NETs (NETosis): disassembly of the cytoskeleton, endoplasmic reticulum vesiculation, chromatin decondensation, plasma membrane and nuclear envelope permeabilization, nuclear lamin meshwork and nuclear envelope rupture to release DNA to the cytoplasm, and plasma membrane rupture and expulsion of DNA to the extracellular environment. (**a**) The low content of lamin A/C in neutrophils allows them to distort their nuclei and pass through narrow spaces during transendothelial migration. (**b**) During NETosis, low lamin A/C content facilitates nuclear envelope rupture. This event also involves nuclear transport of cytoplasmic granule proteins, and low lamin A/C might be also facilitate in this process by easing the control of transport across nuclear pore complexes. GM-CSF, granulocyte-macrophage colony-stimulating factor; VCAM-1, vascular cell adhesion molecule 1; TLR, toll-like receptor; PAMPs, pathogen-associated molecular patterns; DAMPs, damage-associated molecular patterns; CXCL8, C-X-C motif chemokine ligand 8; CXCR, CXC chemokine receptor; ER, endoplasmic reticulum; NE, nuclear envelope; Cyt., Cytoskeleton.

A key property of neutrophils is their ability to traverse narrow constrictions, and cell stiffness results in neutrophil retention in capillaries and arteries [57] and their accumulation in postcapillary venules, leading to vascular inflammation [58]. Nuclear stiffness is promoted during the differentiation of embryonic stem cells through changes to the composition of the nuclear envelope and chromatin structure that result in decreased transcriptional plasticity. In contrast, during the differentiation of promyelocytes into mature neutrophils (known as granulopoiesis), changes in the nuclear envelope generate the characteristic lobed nuclei of neutrophils, which increases their ability to pass through narrow spaces during transendothelial migration and tissue infiltration [27,28]. This lobed nuclear shape is linked to the almost complete loss of lamin A/C expression and increased expression of the lamin B receptor (LBR), another nuclear envelope component [18,28,59,60,61,62]. These changes result in a highly flexible nucleus, facilitating transendothelial migration and passage through narrow tissue spaces [28,63]. Moreover, the lamin A/C content of the nuclear envelope has a stronger influence than nuclear morphology on the ability of neutrophils to pass through constrictions [64] (Figure 2a). Further research into the role of lamin A/C in the molecular basis of cellular and nuclear deformability will advance our understanding of the mechanical aspects of cell biology, and possibly contribute to new therapeutic approaches.

Stimulated neutrophils trap and immobilize pathogens by releasing NETs—web-like DNA-based structures containing histones and cytotoxic antimicrobial proteins [65,66,67]. This process, called NETosis, also has harmful consequences, including tissue damage during sepsis [68,69] and thrombosis [70] and is linked to autoimmune diseases [68]. Before NETosis, the neutrophil nucleus changes its morphology from multilobed to spherical [27]. NETosis engages several cellular mechanisms, including disassembly of the actin, microtubule, and vimentin cytoskeletons; shedding of the plasma membrane; endoplasmic reticulum vesiculation; chromatin decondensation; plasma membrane and nuclear envelope permeabilization; nuclear lamin meshwork and nuclear envelope rupture to release DNA into the cytoplasm; and plasma membrane rupture and expulsion of extracellular DNA. The permeabilization and rupture of the nuclear lamin meshwork generates a punctate lamin A/C meshwork at the nuclear periphery that disassembles to allow DNA to expand throughout the cytoplasm [71]. The reduced lamin A/C content in activated neutrophils might facilitate nuclear envelope rupture, chromatin condensation, and NETosis [27] (Figure 2b). Further experiments are needed to fully define the specific role of lamin A/C in NETosis.

The reduced levels of lamin A/C in neutrophils may also be related to the short lifetime of these proteins [72]. Reduced lamin A/C levels are associated with increased cell fragility [73] and leave neutrophils unprotected against nuclear stress and vulnerable to cell death [74]. The low lamin A/C content of neutrophil nuclei thus appears both to permit the high speed of neutrophil migration and the short lifespan of these cells [75,76].

In the initial phases of NETosis, the neutrophil nucleus imports cytoplasmic granule proteins such as myeloperoxidase and the proteases neutrophil elastase and SerpinB1. Nuclear import of these proteins seems to be important for chromatin condensation and for the antimicrobial function of NETs [77,78,79,80]. The clustering and distribution of nuclear pore complexes and the entry of proteins into the nucleus are regulated by lamin A/C [81], suggesting a possible role for lamin A/C in the trafficking of these proteins during NETosis (Figure 2b). However, no effect of lamin A/C has been reported on nuclear pore complex localization in the neutrophil nuclear envelope during NETosis.

### 4.2. Monocytes/Macrophages

The mononuclear phagocytic system comprises resident macrophages, DCs, circulating monocytes, and monocyte-derived DCs and macrophages, [82]. These cells take part in phagocytosis, inflammation, anti-pathogen responses, and tissue homeostasis [83]. Tissue-resident macrophages are present in all body tissues and may have their origin in the embryonic yolk sac, such as brain microglia, or in the fetal liver, such as lung alveolar macrophages and Kupffer cells in the liver [84,85,86]. Circulating monocytes originate in the bone marrow, and in the steady state these cells are usually found in blood, bone marrow, and spleen [87]. Circulating monocytes are activated and recruited to tissues by several inflammation-induced chemotactic factors such as colony-stimulating factor-1 (CSF-1), chemokine (C-C motif) ligand (CCL)2, CCL5, chemokine (C-X3-C motif) ligand (CX3CL)1, chemokine (C-X-C motif) ligand (CXCL)12, growth factors like vascular endothelial growth factor (VEGF), and even extracellular components such as hyaluronic acid (HA) [86].

Macrophages are classically classified as M1 or M2 macrophages [88,89,90]. M1 differentiation is promoted by activation of TLRs and exposure to Th1 cytokines, including TNFα and interferon (IFN)-γ. The M1 macrophage phenotype is related to the secretion of proinflammatory factors such as TNF-α, IL-1α, IL-1β, IL-6, IL-12, IL-18, IL-13, IL-23, and the chemokines CXCL9, CXCL10, and CXCL11. M1 macrophages also release ROS and nitric oxide (NO) and stimulate the Th1 and Th17 adaptive immune response through antigen presentation [91,92,93,94,95]. The formation of different M2 macrophage subsets is favored by the presence of IL-4, IL-10, IL-13, and TGF-β, among other factors. Most M2 macrophage subsets produce large quantities of immunosupressive cytokines such as IL-10 and TGF-β [96,97].

**Figure 3:**
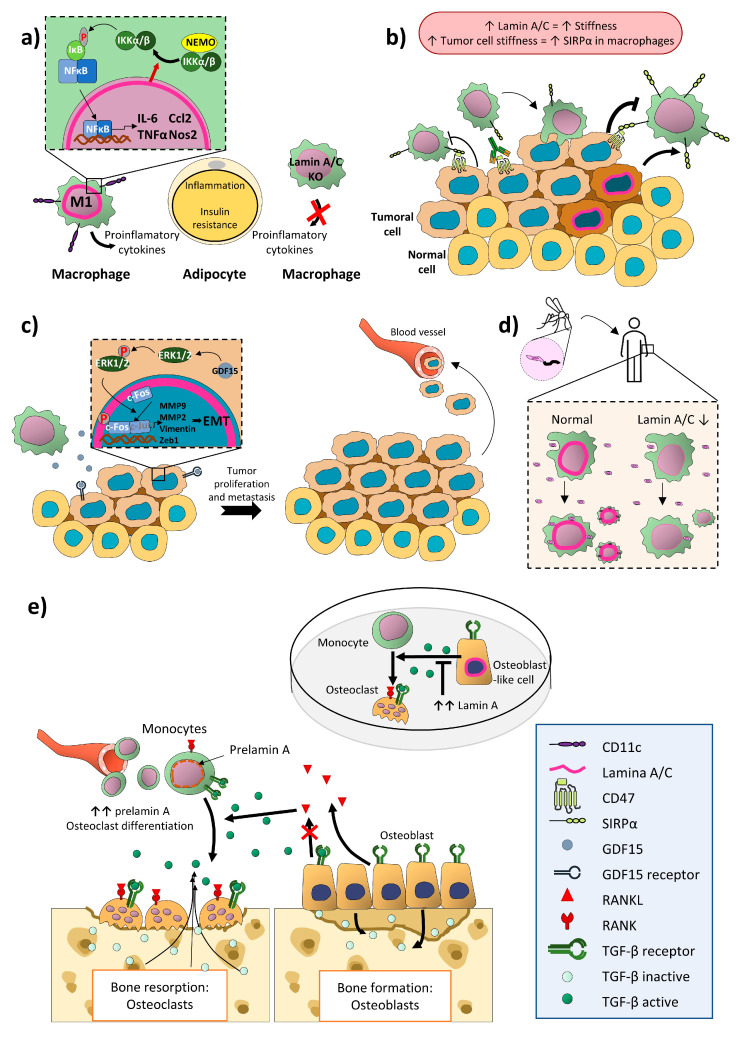
Lamin A/C plays important roles in monocytes and macrophages in pathogen responses, cancer, and obesity. (**a**) Lamin A/C in M1 CD11c+ macrophages promotes obesity-induced insulin resistance and reduces adipose tissue inflammation by activating IKK, promoting NF-κB nuclear translocation, and increasing the expression of proinflammatory genes. (**b**) Expression of CD47 on tumor cells inhibits the action of macrophages by interacting with SIRPα. Lamin A/C content positively correlates with cell stiffness, and stiffness of solid tumors increases macrophage expression of SIRPα, reducing their phagocytic phenotype. The lamin A/C content of tumor macrophages may serve as an index of SIRPα content, potentially providing a means of predicting the efficiency of CD47- SIRPα checkpoint-directed immunotherapies in some cancer types. (**c**) Tumor macrophages produce GDF15, which in colorectal cancer cells releases c-Fos from inhibition by lamin A/C via ERK1/2 phosphorylation, resulting in transcription of AP1-regulated genes and subsequent tumor cell proliferation and metastasis. (**d**) Macrophages are important for resolving infection by *Leishmania braziliensis*, but can also be infected and serve as a niche for parasite replication. Lower lamin A/C content in macrophages diminishes the prevalence of *L. braziliensis* in these cells. (**e**) Bone mass remodeling depends on osteoblasts and osteoclasts. TGFβ stimulates monocytes to form osteoclasts, which mediate bone resorption, and indirectly limits further osteoclast formation by downregulating the osteoclast differentiation factor RANKL in osteoblasts. TGFβ thus regulates bone resorption by first promoting osteoclast differentiation from monocytes and then limiting the extent and duration of resorption. Increased accumulation of the lamin A precursor prelamin A in monocytes induces differentiation towards osteoclasts. In the osteoblast-like cell line U2-OS, Lamin A negatively modulates TGFβ 2 release, which may affect monocyte-to-osteoclast differentiation. T arrow, inhibition; red cross, blockade; up arrow, increase; down arrow, decrease.

Adipose tissue macrophages are the most diverse cell subset of adipose tissue leukocytes and are major regulators of tissue inflammation [98,99]. Obesity-induced low-grade chronic inflammation promotes progression to insulin resistance and type 2 diabetes [99,100], and M1 and M2 macrophages play important roles in these processes as contributors and reducers, respectively [99,101,102]. Mouse models of obesity show strong upregulation of lamin A/C in CD11c+ M1 adipose tissue macrophages. Lamin A/C overexpression in cultured macrophages spontaneously activates IKK; promotes NF-κB nuclear translocation; increases the expression of proinflammatory genes such as *Il6*, *Tnf*, *Ccl2*, and *Nos2*; and activates adipose tissue macrophages. Moreover, myeloid-cell-specific lamin A/C depletion ameliorates obesity-induced insulin resistance and reduces adipose tissue inflammation, suggesting that macrophage-expressed lamin A/C contributes to the development of obesity-induced inflammation and insulin resistance [103] (Figure 3a).

Other cell types interact with macrophages through cell-surface expressed CD47, which binds to SIRPα on macrophages, helping to prevent the phagocytosis of self-cells [104,105]. Several types of cancer cells overexpress CD47 [106,107], thus helping them to inhibit macrophage action and evade the action of the immune system [108]. Several immunotherapies are aimed at blocking this checkpoint to promote the macrophage immune response against tumor cells [109]. Lamin A, but not lamin B, has been described as a mechanosensitive protein [110] whose expression reflects cellular stiffness. The stiffness of solid tumors is related to increased SIRPα expression on macrophages, reducing their phagocytic capacity [108]. Lamin A/C expression in tumor macrophages may thus reflect levels of SIRPα and might predict the efficiency of immunotherapies targeting the CD47-SIRPα checkpoint in individual patients [109] (Figure 3b).

Cancer development, invasion, and metastasis are influenced by the inflammatory microenvironment [111], as is illustrated by the association of chronic inflammation with the development of colon cancer [112,113,114] and its metastasis to liver and lung [115,116]. The activator protein 1 (AP-1) family transcription factor c-Fos regulates cell proliferation, differentiation, invasion, and metastasis [117,118]. c-Fos can be regulated by fast transcription [119] but also by lamin A/C-mediated sequestration to the nuclear periphery, blocking access to its target DNA sequences [120]. This inhibition can be reversed by Erk1/2-dependent c-Fos phosphorylation, which releases c-Fos from lamin A/C, allowing it to bind its DNA sequences and regulate target genes [5,121].

Tumor-associated macrophages infiltrate tumors and produce inflammatory cytokines and chemokines [122], including growth differentiation factor 15 (GDF15), a member of the human transforming growth factor-β (TGF-β) superfamily [123,124,125]. Macrophage-secreted GDF15 promotes colon-cancer-cell invasion and metastasis by inducing ERK1/2-dependent phosphorylation of c-Fos and its release from lamin A/C. This promotes the transcription of AP-1-regulated genes, including *MMP9*, *MMP2*, *Vimentin*, and *Zeb1*, which are implicated in epithelial-mesenchymal transition [126] (Figure 3c).

Macrophages are also the targets of infection by *Leishmania* parasites. Leishmaniasis is caused by protozoan parasites of the *Leishmania* genus, including the species *L. braziliensis*. *Leishmania* parasites alternate between two major forms, the free-living flagellated promastigote found in phlebotomine sandfly vectors and the obligate intracellular aflagellated amastigotes in vertebrate phagocytic cells, mainly macrophages [127,128]. Infection provokes a rapid infiltration of neutrophils, which phagocytose most of the parasites and produce chemokines and cytokines to recruit and activate other leukocytes. However, neutrophils do not completely eliminate the infection and die by apoptosis. The apoptotic neutrophils are internalized by macrophages and dendritic cells, where the parasite divides, increasing and dispersing the infection. Macrophages thus have a dual effect on *Leishmania* infection, since they contribute to the eradication of internalized parasites but also provide a safe place for *Leishmania* replication [129,130]. The proportion of human macrophage U937 cells infected with *L. braziliensis* is reduced by shRNA silencing of lamin A/C, and the lamin A/C silencing also reduces the number of parasites per sampled macrophage, suggesting that lamin A/C plays a role in the prevalence of *Leishmania* parasites in macrophages [131]. Further experiments are needed to corroborate these results in primary macrophages and DCs and to discern how lamin A/C affects parasite eradication and division in macrophages (Figure 3d).

Experiments in disheveled hair and ears allele Lmna (Lmna^Dhe^) mice also implicate lamin A/C in the response of macrophages to viral infection. Lmna^Dhe^ mice carry the spontaneous amino acid substitution L52R in the Lmna gene, and are characterized by accelerated aging, epidermal dysplasia, and craniofacial defects [132]. These mice show increased susceptibility to infection after intranasal inoculation with the Influenza A virus. The elevated mortality and higher viral burden after influenza infection in these mice is paralleled by substantial immune-cell shifts, including reduced accumulation of lung alveolar macrophages, systemic expansion of immune suppressive Foxp3+ Tregs, and immune dominance of viral-specific CD8+ T cells [133], suggesting that lamin A/C plays an important role in the response of macrophages and T cells to viral infection. Peripheral macrophages from Lmna^Dhe/−^ mice show increased production of inflammatory factors such as NK-κB and TNFα, linking increased inflammation triggered by defective lamin A/C function in macrophages to the origin of otitis media and hearing deficits in these mice [134].

Human immunodeficiency virus type 1 (HIV-1) infects nondividing cells [135], preferably crossing the nuclear envelope of mature monocyte-derived macrophages than immature monocyte to enter the nuclei [136,137,138]. The infection of terminally-differentiated macrophages, among other cell types, serves at the initial steps of infection and as virus reservoirs [135]. In this process, the viral protein the auxiliary viral protein R (Vpr), a member of the preintegration complex (PIC) of HIV-1, arrests the cell cycle at the G2/M transition and induces temporary, localized herniations in the nuclear envelope, linked to alterations in the nuclear lamina [139]. Moreover, lamin A/C tethered the inner nuclear membrane protein Sad1 and UNC84 domain containing 2 (SUN2) to the nucleosomes 1 and 2 promoting the repressive chromatin including the HIV-1 5′-LTR an so, repressing the initiation and elongation of HIV-1 transcription, therefore regulating HIV-1 infection and latency [140]. This coincides with the fact that the overexpression of SUN2 inhibits HIV-1 infection in primary monocyte-derived cells [141].

Several other studies have highlighted the importance of lamin A/C for correct macrophage function and the macrophage regulation of the development and invasion capacities of other cells, such as colon cancer cells. Closing the circle, lamin A/C in skeletal muscle, but not in osteoblast-lineage cells, has been shown to control osteoporosis and the differentiation of osteoclasts, the resident macrophages in bone [142], through the production of the proinflammatory cytokine IL-6 [143].

Bone mass remodeling depends on the interaction between osteoblasts and osteoclasts to balance bone formation and resorption. Osteoclasts promote bone resorption by secreting hydrochloric acid and proteases to dissolve the calcified bone matrix. Osteoclasts are generated by the cytokine-driven proliferation and differentiation of monocyte precursors [144]. One of the key cytokines involved is TGFβ, which controls bone remodeling through independent facilitative and suppressive actions on osteoclast differentiation and bone resorption, respectively [145]. TGFβ is produced by osteoblasts in an inactive form and deposited in the bone matrix [146,147,148]. Following the initiation of resorption, TGFβ is released from the bone matrix [149]. This released TGFβ stimulates monocytes to form osteoclasts instead of following macrophage differentiation pathways and also acts on osteoblasts to reduce expression of the osteoclast differentiation factor RANKL (receptor activator of NFκB ligand) and thereby indirectly limit further osteoclast formation. TGFβ thus regulates bone resorption by first steering monocytes to differentiate into osteoclasts and then subsequently limiting the extent and duration of resorption [145].

Differentiation of monocytes towards osteoclasts is also promoted by increased accumulation of the lamin A precursor form prelamin A [150]. Prelamin A undergoes a series of posttranslational modifications and a final proteolytic cleavage to generate mature lamin A [2,144], and abnormal accumulation of prelamin A underlies some human diseases called laminopathies [2]. Osteoblasts from patients with mandibuloacral dysplasia (MADA), a laminopathy produced by LMNA mutations, secrete excess amounts of TGFβ 2, which in turn triggers differentiation of monocytes into osteoclasts [151]. Moreover, wild-type lamin A negatively modulates TGFβ 2 release in osteoblast-like human U2-OS osteosarcoma cells [151], with possible effects on monocyte to osteoclast differentiation [145]. 

### 4.3. Dendritic Cells

**Figure 4:**
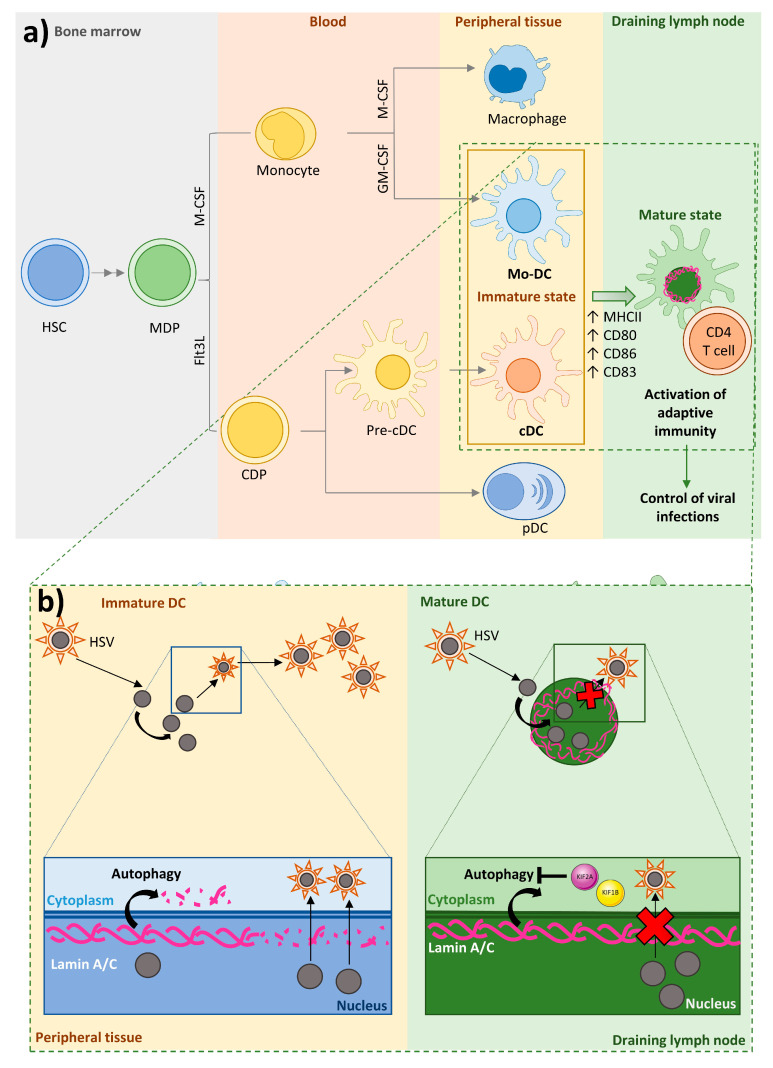
DC ontogeny and the importance of DC-expressed lamin A/C in the response to HSV infection. (**a**) A common progenitor, the macrophage/dendritic cell progenitor (MDP), gives rise to macrophages, monocyte derived-DCs, conventional DCs, and plasmacytoid DCs. (**b**) DCs play an important role in the initiation of immune responses against pathogens through their function as professional APCs and their secretion of cytokines. Depending on their state of maturation, DCs are located in peripheral tissues (immature DCs) or in draining lymph nodes (mature DCs), to where they migrate after encountering and taking up antigens. In draining lymph nodes, DCs stimulate adaptive immune responses that help to control and destroy viral infection. Some viruses infect DCs, promoting the production, release, and spreading of viral particles in host cells and the host organism. In the case of herpex simplex virus (HSV), nuclear egress of progeny capsids, the formation of new infectious particles, and the viral release and spread depend on the nuclear lamina permeabilization and lamin A/C degradation by autophagy. Unlike immature DCs, mature DCs have an elevated protein content of the mTOR activators KIF1B and KIF2A, leading to inhibition of autophagy-dependent lamin A/C degradation after HSV infection and thus limiting viral nuclear egress, viral particle formation, and virus release and spread.

DCs provide a link between innate and adaptive immunity by presenting antigens to and activating T cells. A common dendritic progenitor (CDP) gives rise to several DC subtypes. CDPs generate plasmacytoid DCs (pDCs) in bone marrow, pre-DCs that circulate in the blood, and classical DCs (cDCs) in lymphoid and nonlymphoid organs [152,153,154,155,156]. The generation of pDCs versus cDCs depends on the growth factor fms-like tyrosine kinase 3 ligand (Flt3L) [157]. Monocytes originate in the bone marrow and circulate in blood. Circulating monocytes migrate to inflamed tissues to generate macrophages or monocyte-derived DCs, in response to the growth factors M-CSF or GM-CSF, respectively. Monocyte-derived DCs have a greater APC capacity than monocytes [158,159]. Through their function as professional APCs and their ability to secrete cytokines, DCs play important roles in initiating immune responses to invading pathogens; cDCs and monocyte-derived DCs are potent APCs, whereas pDCs specialize in secreting type I interferon (IFN-I) [160] (Figure 4a).

Immature DCs reside in peripheral tissues under steady state conditions, where they encounter and take up antigens [161]. Upon activation, DCs increase the expression of major histocompatibility complex molecules (MHC) class II (MHCII) and the costimulatory molecules CD80, CD86, and CD83 [156,160] and migrate along a chemokine gradient to draining lymph nodes, where they enter paracortical T cell zones to activate and prime antigen-specific naïve T cells and secrete cytokines [162,163,164,165]. In this way, DCs stimulate adaptive immune responses that help to control and eliminate invading viruses [166,167]. However, some pathogens infect DCs, thus subverting the immune system and spreading infection in the host [166,167]. This is the case of Herpes simplex virus type 1 (HSV-1), a member of the α-herpesvirus family that persists in a latent state in sensory neurons and ganglia after primary infection and can lead to severe local and systemic disease in immunosuppressed patients [168,169].

HSV-1 replicates in the nucleus, where viral capsids form before their transport across the nuclear envelope into the cytoplasm for secondary envelopment. The nuclear lamina hinders the nuclear egress of viral capsids [170]. In several cell types HSV-1 disrupts the nuclear lamina by phosphorylating lamin A/C via the action of the viral protein kinase US3 [171,172,173]. However, in immature DCs, HSV-1 infection triggers the degradation of lamin A/C and other lamins by autophagy [174], a mechanism upregulated by HSV-1 infection [175], thereby facilitating the transfer of viral caspids across the nuclear envelope independently of lamin A/C phosphorylation [174]. HSV-1 infection of immature DCs results in a fast lytic replication cycle and the release of high numbers of progeny virions [176]. In contrast, infection of mature DCs is blocked after early gene expression, resulting in a limited progeny [177], despite the fact that immature and mature DCs both produce HSV-1 proteins upon infection [178]. The resistance of mature DCs to viral replication is due to their elevated expression of the kinesin family proteins KIF1B and KIF2A. These proteins promote mTOR activity, which in turn inhibits autophagy [179], likely by hampering autophagosome-lysosome fusion, thus blocking lamin A/C degradation. This inhibition of lamin A/C degradation in mature DCs limits the nuclear egress of progeny viral capsids and thus the formation of new infectious particles, blocking release of viral particles and their spread in the host organism [174]. DC-expressed lamin A/C thus plays a key role in the immune response against viral infections (Figure 4b). Further experiments are needed fully define the mechanisms of nuclear protein degradation via autophagy [180].

Some migrating cells express low amounts of lamins, whereas others release proteases to create pores in the extracellular matrix to pass through small pores [181]. Protease-mediated matrix degradation is used by metastatic tumor cells, but appears to be less used by migrating immune cells [182,183]. In neutrophils, the low lamin A/C content seems to increase the ability of cells to pass through small pores [64] but also reduces cell viability [74]. However, DCs need to combine the ability to migrate with long-term survival, in order to enable them to patrol peripheral tissues and migrate to draining lymph nodes for antigen presentation to T cells [32]. To meet this need, DCs accumulate perinuclear actin nucleated by Arp2/3 downstream of Wave2; this perinuclear actin deposition allows the cells to deform the nucleus and pass through constrictions, probably by disrupting the nuclear lamina. Arp2/3-dependent perinuclear actin nucleation is necessary for migration only in lamin A/C-expressing cells, suggesting that DCs must transiently rupture or disassemble the lamin A/C meshwork to allow them to pass through constrictions [32]. 

## 5. Lamin A/C in Adaptive Immunity

Adaptive immunity is a specific and enduring immune response that helps to distinguish foreign from self-antigens. Adaptive immunity is mainly mediated by T and B cells, which accurately distinguish self- from nonself-antigens via the T cell receptor (TCR) and B cell receptor (BCR), respectively [184]. T cells are categorized according to their membrane and intracellular markers. They express the αβ or γδ TCR, CD3, and one of the coreceptors CD4 or CD8. The TCR-CD3 complex recognizes antigens presented in the context of MHC molecules (human leukocyte antigen [HLA] in humans) by an APC [184]. B cells generate antibodies, act as APCs, and produce cytokines. B cells express the cell-lineage marker CD19 in addition to surface and intracellular proteins and the distinct immunoglobulin BCRs [184].

APCs are able to present antigens to cognate naïve CD4+ and CD8+ T cells [185]. Upon antigen recognition, CD4+ T cells activate, proliferate, and differentiate into specialized effector T helper (Th) cells, whereas CD8+ T cells activate, proliferate, and differentiate into CD8+ cytotoxic T lymphocytes (CTL) [186,187]. Naïve CD4+ T cells can differentiate into several cell subtypes, depending on the type of antigen encountered, the TCR signal intensity, and the local cytokine milieu [19,188,189,190]; these subtypes include effector T cells (T helper 1 [Th1], Th2, and Th17) and regulatory T cells (Treg) [189,191]. Th1 cells are distinguished by the production of large amounts of IFN-γ and IL-2 and the production of the master transcription factor T-bet. Th2 cells release IL-4, IL-5, and IL-13 and express the master transcription factor GATA-3. Th17 cells produce the cytokine IL-17 and are defined by expression of the transcription factor RORγt [192]. Tregs are characterized by the expression of the transcription factor forkhead box 3 (Foxp3) and the protein CD25. CD8+ T cells are responsible for defense against intracellular pathogens and recognize antigen via the TCR in the context of ubiquitously expressed MHC class I molecules (MHC-I; HLA-A, -B, or -C in humans). CD8+ T cells exert their role through the release of proinflammatory cytokines [187] such as TNF-α, and IFN-γ. CD8+ T cells stimulate Fas-receptor-mediated apoptosis of the target cell via the production of Fas ligand and target-cell lysis via the production of granzymes and perforin. [193]

T cell activation is triggered by the recognition of an antigen presented by APCs in the context of MHCII for CD4+ T cells or MHCI for CD8+ T cells [186,187]. In this process, T cells and APCs form specialized cell-cell contacts called immunological synapses (IS). The IS is a highly organized structure that enables efficient transient cell-cell communication [194,195]. IS formation and maintenance depends on the modulated recruitment of membrane receptors to specific subcellular localizations. The TCR and CD3 accumulate at the central supramolecular activation cluster (cSMAC), and actin, integrins, and other adhesion molecules accumulate at the surrounding peripheral SMAC (pSMAC). T cell activation also necessitates the movement of the microtubule organizing center (MTOC) towards the T cell-APC contact site. The relocated MTOC then guides the polarization of intracellular vesicles to the IS [185].

In Lmna^−/−^ mice, the lack of lamin A/C induces profound defects in T and B cell development, which have been attributed to indirect effects associated with the loss of lamin A/C in nonimmune cells [196]. Moreover, splenocytes and CD4+ T cells isolated from spleens of Lmna^−/−^ mice show impaired activation in response to a variety of TCR-dependent stimuli [16,19]. Experiments with a hapten-induced contact hypersensitivity (CHS) mouse model [197] revealed that lamin A/C proteins modulate the immune response in vivo. Reconstitution of irradiated wild-type mice with bone marrow cells from Lmna^−/−^ mice reduced the severity of ear inflammation in the CHS model. The adoptively transferred wild type animals also had a reduced content of Lmna^−/−^ CD4+ T cells in ears, lymph nodes, and spleen [16]. Loss of lamin A/C in CD4+ T cells did not affect T cell proliferation [190], a result that may reflect the transient nature of lamin A/C expression in T cells after antigen recognition, since lamin A/C has been implicated in the control of proliferation in fibroblast [198,199]. Alternatively, the transient expression of lamin A/C in activated T cells might be related to the need for these cells to later exit the draining lymph nodes and migrate to target tissue.

**Figure 5:**
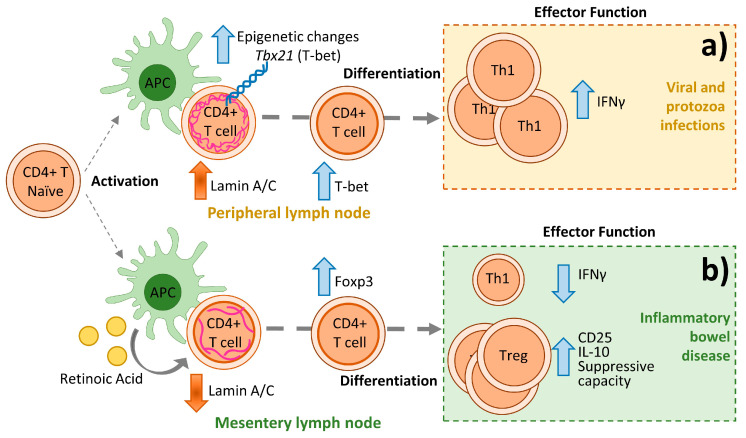
Lamin A/C in CD4+ T cells respectively promotes and inhibits the differentiation and function of Th1 cells and Tregs. Upon antigen recognition, CD4+ T cells activate, proliferate, and differentiate into specialized effector T helper (Th) cells. (**a**) In the context of viral or protozoan infection, lamin A/C expression increases CD4+ T cell Th1 differentiation by epigenetically regulating the mRNA expression of Tbx21 (T-bet). (**b**) In inflammatory bowel disease, the absence of lamin A/C in CD4 T cells enhances FOXP3 expression, which is reflected in increased Treg differentiation and function, important in resolving the impaired Th1 response. Vertical arrows indicate changes in expression or function.The efficacy of anti-tumor drugs depend on their capacity to induce apoptosis since apoptosis evasion is a redundant characteristic of cancer cells [223]. During apoptosis, the nuclear envelope is permeabilized by rupture of nuclear pore complex and nuclear lamina proteins, including lamin A/C [224]. Classical Hodgkin’s lymphoma is a kind of B cell lymphoma contained of Hodgkin and Reed-Sternberg cells with a unique or multiple nucleus, respectively [17]. Hodgkin and Reed-Sternberg cells show lamin A/C expression [33] and impaired lamin A/C structure, which is dissimilar from unstimulated common lymphocytes [17]. It has been described a caspase-dependent and independent [224] cleavage of lamin A/C, which dismantles the nuclear lamina, and promotes apoptosis in B cell lymphomas [225,226].

T cells coordinate the defense against microbial pathogens [189]. Differentiation into distinct specialized effector Th cells is crucial for eliciting an appropriate host defense that triggers immune-mediated inflammation without causing tissue injury [186]. Th1 differentiation, required for host defense against intracellular pathogens, involves IL-2-dependent production of IFNγ mediated by the transcription factor T-bet [200]. Th2 differentiation is triggered by extracellular pathogens or allergens through the production of GATA-3 and the IL-4-dependent activation of STAT6 [201]. Lamin A/C expression promotes CD4+ T cell Th1 differentiation in response to viral and protozoan infections by regulating T-bet expression and IFNγ production [202]. The absence of lamin A/C in CD4+ T cells inhibits ERK1/2 signaling [202], which is associated with Th1 differentiation [188,203], and epigenetically downregulates the expression of T-bet [44]. Epigenetic regulation of the T-bet gene *Tbx21* by lamin A/C in CD4+ T cells seems to be mediated at least in part by EZH1 [44], a component of the epigenetic-modifying polycomb complex PRC2 [204,205] (Figure 5a). These results confirm that epigenetic regulation is a key determinant of Th fate [206] and that lamin A/C is an important epigenetic modulator of T cell differentiation [207,208].

Natural Tregs, which originate in the thymus, mostly intervene in the acceptance to self-antigens through the production of IL-10 and TGF-β [209]. Inducible Tregs (iTregs) are highly involved in pathogen tolerance and are generated from naïve T cells in the periphery [210]. Tregs play a significant role in self-tolerance, the inhibition of immune responses, and homeostasis [211] by regulating the immune balance. Treg differentiation and function is triggered by CD11c+ CD103+ DCs [212] and signaling from IL-10 and TGF-β [213] through the induction of Foxp3 expression [189]. Tregs inhibit CD4+ and CD8+ T cells, B cells, and NKT cells and conduct DCs and macrophages to a more tolerogenic phenotype through numerous mechanisms [214,215,216].

Tregs modulate cells through contact-dependent cell-mediated inhibition mediated by surface expression of suppressive molecules such as T lymphocyte-associated antigen-4 (CTLA-4) and programmed cell death-1 (PD-1). CTLA-4 downregulates APC function and T cell activation by reducing CD80 and CD86 membrane expression in the APC and by blocking the co-stimulatory interaction between CD80/CD86 on the APC and CD28 on the T cell [217]. Tregs can promote effector T cell eradication by releasing granzyme B [218] or by producing the tumor necrosis factor-related apoptosis inducing ligand 2 (TRAIL2; also known as death receptor 5 [DR5]) and galectin-1 [219,220]. Tregs also secrete the immunosuppressive cytokines TGF-β, IL-10, and IL-35 [214]. The absence of lamin A/C in CD4+ T cells enhances FOXP3 expression, reflected in increased Treg differentiation and function [44]. Tregs play a crucial role in resolving the impaired Th1 and Th17 response that mediates bowel inflammation in inflammatory bowel disease (IBD), a chronic inflammatory disease of the intestine that includes ulcerative colitis and Crohn disease [221,222]. Lamin A/C deficiency in CD4 T cells protects against IBD by diminishing Th1 differentiation and facilitating Treg differentiation and function [44] (Figure 5b).

## 6. Discussion

Recent advances suggest that lamin A/C is finely regulated in immune cells, in both the level and the time window of expression. Lamin A/C is highly expressed, and the performance of their cell functions requires macrophages to increase its expression, while neutrophils decrease expression. This regulation appears to be related to the specific necessities of these cells types, with increased lamin A/C expression in macrophages potentially related to the exertion of proinflammatory functions and long lifespan, whereas lamin A/C inhibition in neutrophils facilitates migration while reducing lifespan. Lamin A/C expression in DCs seems to be intermediate between macrophages and neutrophils, allowing more efficient and faster migration than macrophages, and longer viability than neutrophils. T cells are a case apart, with transient lamin A/C expression increasing cellular activation and direct differentiation toward a more proinflammatory Th1 phenotype, while decreased expression appears to have no effect on proliferation and probably migration. Overall, increased lamin A/C expression appears to be compatible with higher cell activation, as observed in naïve T cells upon antigen recognition and macrophages upon LPS stimulation. The importance of lamin A/C expression for cell activation and its microenvironment-dependent regulation in activated T cells suggest that this regulation might be reproduced in other immune cell types, pointing to possible future research directions.

Lamin A/C intervenes in a plethora of functions in immune cells, with prominent effects on migration, nuclear envelope permeability to pathogens, pathogen dispersal, cytokine production, and mechanosensitivity. Lamin A/C is consequently implicated in many cellular and biochemical processes associated with disease. This relevance extends beyond laminopathies, with lamin A/C showing involvement in the immune response to viruses and protozoa through its functions in macrophages and T cells, in cancer through its role in DCs and macrophages, in diabetes and obesity through expression in macrophages, and in osteoporosis through its actions in monocytes. Further research is needed to achieve a comprehensive understanding of the role of lamin A/C in immune cells and immune-mediated human disease.

## Abbreviations

AP-1	activator protein 1
CCL	chemokine (C-C motif) ligand
COPD	chronic obstructive pulmonary disease
CSF-1	colony-stimulating factor-1
cSMAC	central supramolecular activation cluster
CX3CL	chemokine (C-X3-C motif) ligand
CXCL	chemokine (C-X-C motif) ligand
DAMPs	damage-associated molecular patterns
DCs	dendritic cells
GDF15	growth differentiation factor 15
HA	hyaluronic acid
HIV-1	human immunodeficiency virus type 1
HL-60	human promyelocytic leukemia cells
HSV-1	herpex Simplex Virus
Lmna^Dhe^	hair and ears allele Lmna
LPS	lipopolysaccharide
MHC-I	antigen/major histocompatibility complex I
MHC-II	antigen/major histocompatibility complex II
MTOC	microtubule organizing center
NETs	neutrophil extracellular traps
NKT	cells natural killer T cells
NO	nitric oxide
PAMPs	pathogen-associated molecular patterns
PRRs	pattern recognition receptors
pSMAC	peripheral supramolecular activation cluster
RA	retinoic acid
ROS	reactive oxygen species
SUN2	Sad1 and UNC84 domain containing 2
TLRs	toll-like receptors
TPA	phorbol ester
TGF-β	transforming growth factor-β
VEGF	growth factors like vascular endothelial growth factor
Vpr	viral protein R
